# Atomic-Level Structural Engineering of Graphene on a Mesoscopic Scale

**DOI:** 10.1021/acs.nanolett.1c01214

**Published:** 2021-06-09

**Authors:** Alberto Trentino, Jacob Madsen, Andreas Mittelberger, Clemens Mangler, Toma Susi, Kimmo Mustonen, Jani Kotakoski

**Affiliations:** † University of Vienna, Faculty of Physics, Boltzmanngasse 5, 1090 Vienna, Austria; ‡ Nion Company, 11511 NE 118th Street, Kirkland, Washington 98034, United States

**Keywords:** graphene, defect engineering, electron microscopy, machine learning, automation

## Abstract

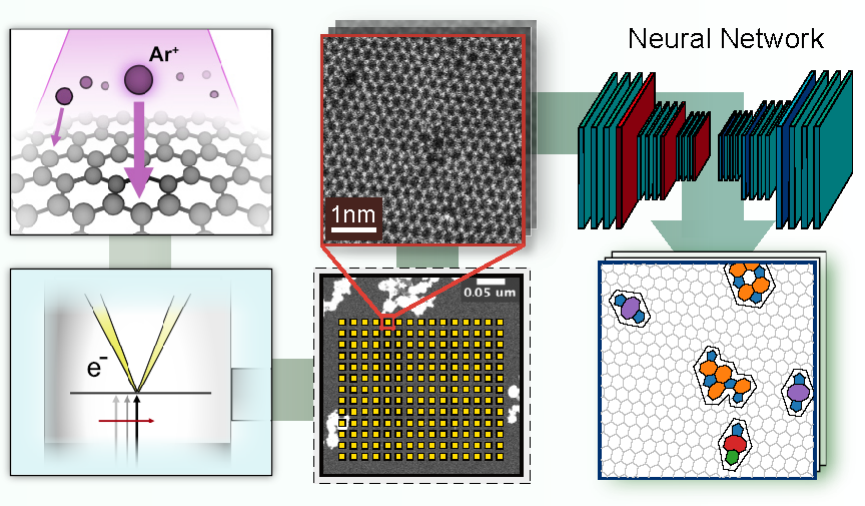

Structural engineering is the first step toward changing properties of materials. While this can be at relative ease done for bulk materials, for example, using ion irradiation, similar engineering of 2D materials and other low-dimensional structures remains a challenge. The difficulties range from the preparation of clean and uniform samples to the sensitivity of these structures to the overwhelming task of sample-wide characterization of the subjected modifications at the atomic scale. Here, we overcome these issues using a near ultrahigh vacuum system comprised of an aberration-corrected scanning transmission electron microscope and setups for sample cleaning and manipulation, which are combined with automated atomic-resolution imaging of large sample areas and a convolutional neural network approach for image analysis. This allows us to create and fully characterize atomically clean free-standing graphene with a controlled defect distribution, thus providing the important first step toward atomically tailored two-dimensional materials.

Aberration-corrected transmission electron microscopy (TEM) is an indispensable tool for materials science. Notably, in the context of 2D materials, modern instruments are able to resolve the position of every atom within the limited field of view of about 100 nm^2^ that is observable at any one time. In the case of light atoms observed using annular dark field (ADF) detectors in scanning transmission electron microscopes, their elemental composition can also be deduced simply based on the imaging contrast.^1^ Therefore, no fundamental reason exists for why properties such as the electrical conductivity or spectroscopic response could not be associated with the exact atomic configuration of 2D materials containing impurities or defects, even up to the level of completely disordered structures.^2,3^ However, until now, the number of local atomic configurations that can be manually recorded and analyzed within reasonable time has limited such analysis to local snapshots that typically remain insufficient for reliable sample-wide extrapolations, and their selection is prone to operator bias. Correspondingly, statistical analysis of defect structures remains rare in microscopy studies. Here, we describe an experimental platform that relaxes these constraints and allows for both large-scale structural engineering as well as analysis of 2D materials down to atomic resolution. We combine interconnected ultrahigh vacuum subsystems comprised of an aberration-corrected scanning TEM (STEM) instrument with automated image acquisition and a microwave plasma generator which we use as an ion source for atomic-scale structural engineering. A convolutional neural network (CNN) is used for identification of the atom positions, their element-specific contrast, and the resulting topology. Graphene,^4^ a one-atom-thick membrane consisting of sp^2^-hybridized carbon atoms in a hexagonal arrangement, provides the ideal benchmarking material for defect-engineering experiments due to its self-healing ability that allows it to retain structural integrity up to a very high vacancy concentration^5^ and even complete amorphization.^6^ In some previous experiments, graphene has been irradiated both with the imaging electrons in a TEM instrument^6^ as well as with energetic ions^7^ to introduce disorder into the structure. In the first case, the modification has been limited by the beam current and size to some hundreds of nm^2^, whereas in the second case the samples have typically suffered from significant hydrocarbon contamination, limiting the observed areas to a small fraction of the sample area.^8,9^ Although these issues can be overcome to some degree by carrying out the experiment in a vacuum system combining an ion gun and an atomic-resolution scanning probe microscope^10,11^ which is common in surface science setups, the imaging of large sample areas still remains a challenge. Nevertheless, such experiments combined with Raman spectroscopy have thus far provided the best way to characterize disordered graphene structures.^12^ However, bridging the gap between images of individual defect structures and the length scale probed with Raman spectroscopy (some hundreds of nanometers in diameter, with the exception of tip-enhanced techniques) requires assumptions of the size, spatial distribution, and type of the defects. This can be overcome only through large-scale atomic-level characterization, such as is demonstrated here.

## Experimental Design

**Figure 1:**
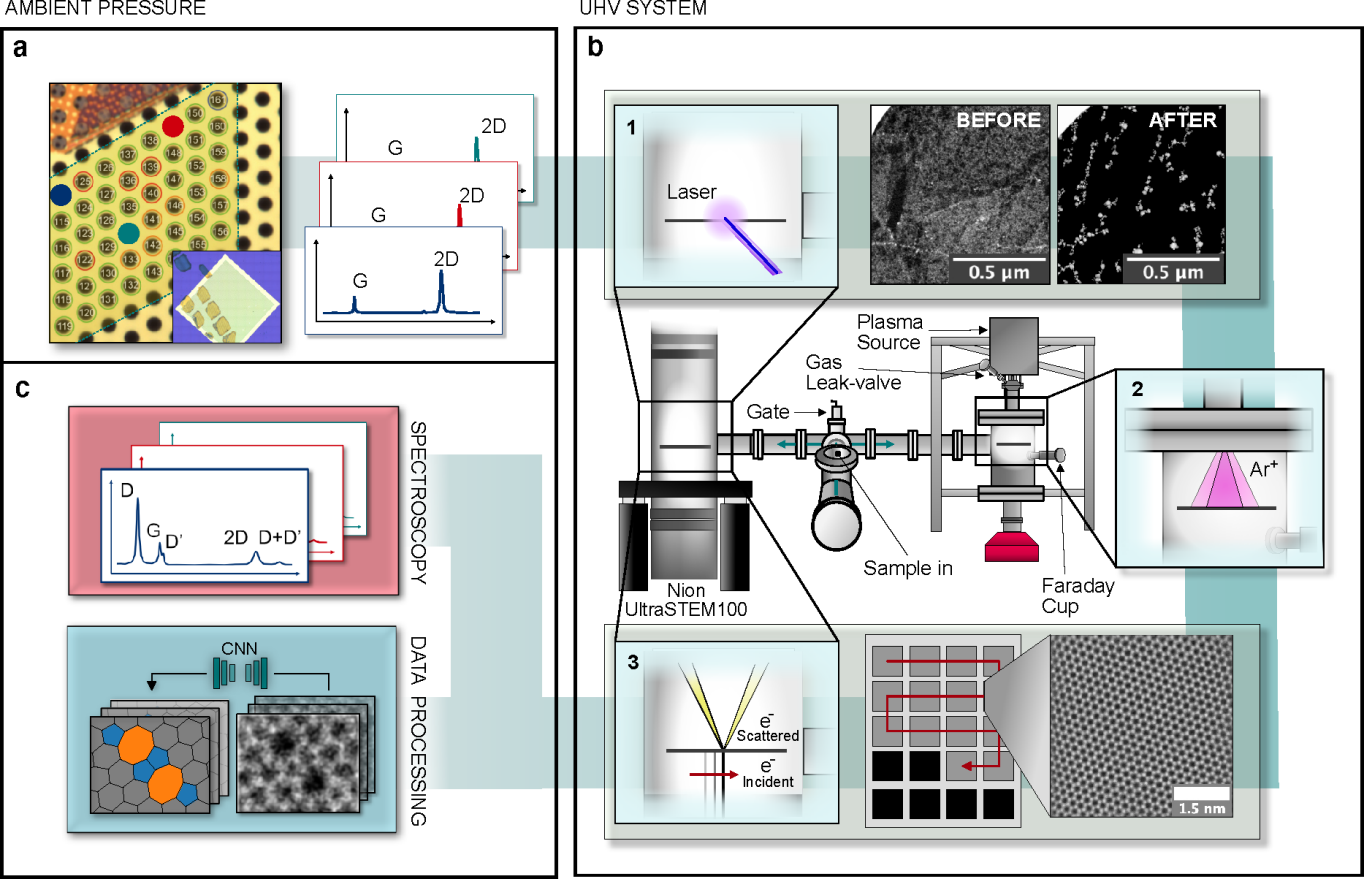
Overview of the experiment. (a) Light microscopy image of a part of the as-prepared graphene sample on the SiN grid suspended over an array of 2.5 μm holes. The inset shows a larger area of the sample with visible polymer residues used for navigating in the different instruments. Each suspended area that was characterized is labeled, and the three holes that were selected for complete analysis are marked with colored circles. (b) After precharacterization, the sample is introduced into the vacuum system, where it is first cleaned in the microscope column with a laser (1); the two STEM-ADF images show the result of the cleaning (dark area corresponds to clean graphene). Next, the sample is moved to the chamber with the plasma source to be irradiated with Ar^*+*^ ions (2). After irradiation, the sample is imaged in the microscope along a serpentine path (3) using an automated image acquisition algorithm. The inset shows an atomic-resolution image of a pristine sample area. (c) The microscopy images are passed to the neural network for analysis. The inset images show examples of the detected topology and the corresponding original image. After imaging, the sample is taken out from the vacuum system, and Raman spectra are again recorded for each hole.

We start our experiments by preparing monolayer graphene (commercial samples grown via chemical vapor deposition) on a perforated SiN substrate with 2.5 μm holes. The initial quality of the sample is assessed via Raman spectroscopy before the sample is inserted into the vacuum system (see Methods and Supporting Information for additional details). This allows selecting sample areas with good coverage as well as not containing excessive amounts of surface contamination^13^ or pre-existing defects (Figure 1a), as illustrated by the absence of peaks within the D-band region of the spectrum. Next, remaining surface contamination is removed by laser irradiation (Figure 1b.1) in the column of the Nion UltraSTEM 100 microscope,^14^ which is also used to confirm through annular dark field (ADF) imaging that atomically clean graphene has been created over a sufficient number of the micrometer-sized holes. Next, the sample is transferred through a vacuum line (with a base pressure of ca. 10^–8^ mbar) to a chamber containing a plasma generator where low-energy Ar^+^ ion irradiation is used to create defects (up to an irradiation dose of 3.2 × 10^13^ ions cm^–2^ as estimated with a Faraday cup) with parameters that are expected to create mainly single and double vacancies^15^ (Figure 1b.2). Simultaneously to the Ar ion treatment, the sample is irradiated with a diode laser which reduces the amount of deposited surface contamination by keeping the sample at an elevated temperature.^16^ After the plasma treatment, the sample is transported back to the microscope for atomic-scale structural analysis (Figure 1b.3). We note that it is of utmost importance that the irradiated materials are not exposed to air prior to characterization in the electron microscope. Air exposure would lead to partial coverage of the sample with environmental contamination, which can lead to significant bias in the results and even completely obscure some atomic configurations. After microscopy, the recorded images are analyzed by the neural network, and the sample is removed from the vacuum system for final characterization via Raman spectroscopy (Figure 1c).

## Sample Cleaning

**Figure 2:**
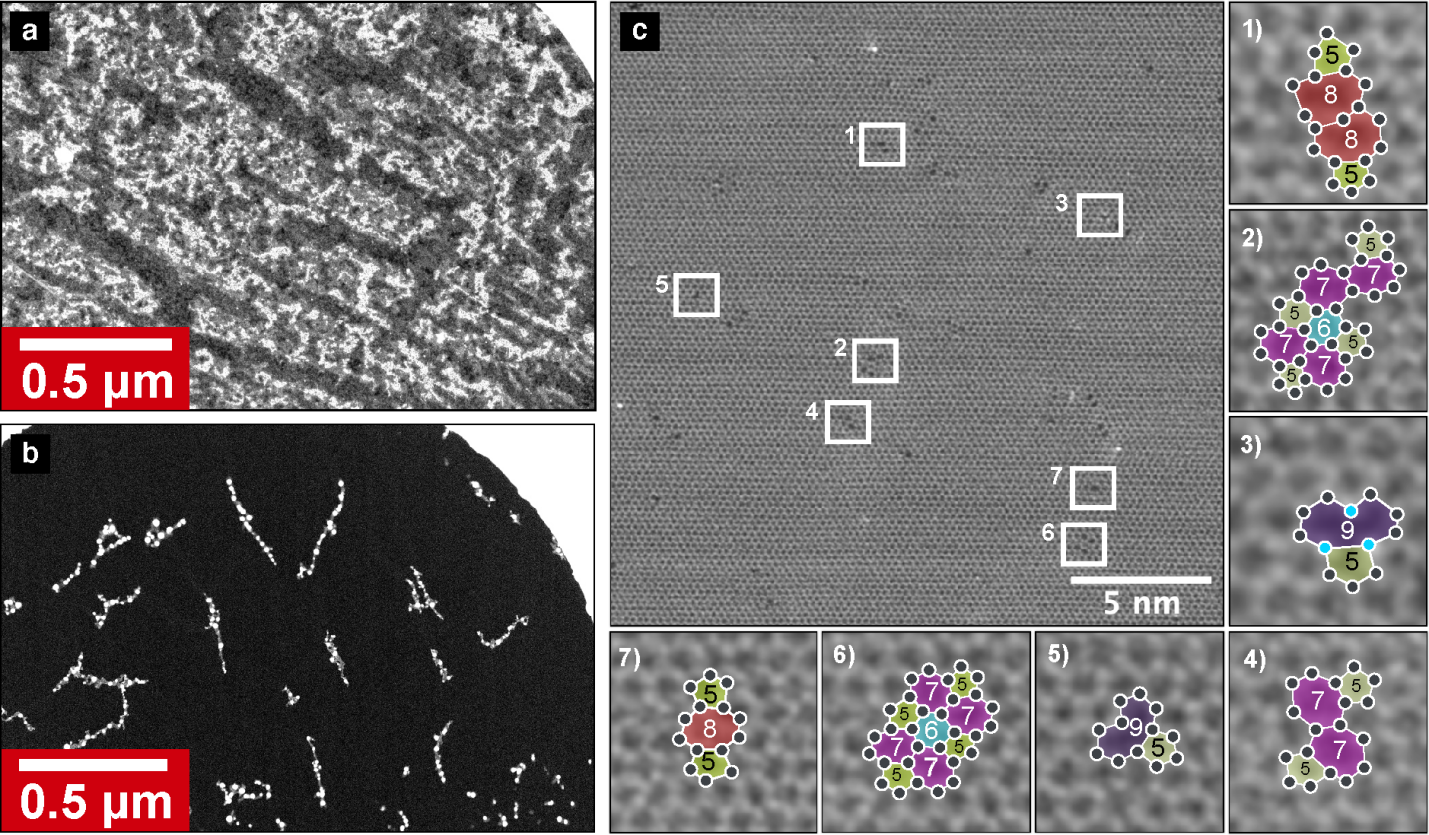
Atomic structure before and after cleaning and ion irradiation. (a) STEM-ADF images of suspended graphene before and after (b) laser cleaning in the microscope column. The entire dark area in (b) corresponds to atomically clean graphene. (c) An example STEM-ADF image of a sample area with a particularly high defect density after Ar^+^ irradiation, demonstrating that even defected areas have remained contamination free during the process. Seven of the defects are further shown at higher magnification to demonstrate the variety of different defect types that can be created during the irradiation process. They include monovacancies *V*_1_(59) (c.3, c.5), a trivacancy *V*_3_(5885) (c.1), divacancies *V*_2_(55–8) (c.7) and *V*_2_(55–77) (c.4), and more complex structures (c.2 and c.6). The light blue dots in c.3 mark possible impurity atoms based on the image contrast.

The main results of preirradiation laser cleaning and Ar^+^ irradiation are shown in Figure 2. Even in the highest-quality areas according to Raman characterization, the as-prepared sample contains significant surface contamination. This contamination contributes most of the contrast seen in Figure 2a. After cleaning (Figure 2b), practically all of the contamination has been removed, and only a few strings of nanoparticles, that based on their contrast consist predominantly of elements heavier than carbon, remain. The laser spot hitting the sample in the setup has the shape of an oblong rectangle with vertices of 0.3 mm and 1.5 mm and deposits ca. 60 mJ of thermal energy during each 100 ms pulse at 600 mW power used for cleaning (see also Supporting Information Figure S6). When necessary, consecutive pulses can be used to clean the observed area entirely. Figure 2c shows atomic-scale images of a clean ca. 25 × 25 nm^2^ area on the sample containing a number of defects created by the Ar^+^ irradiation (note that this image was selected due to the particularly high defect density). The marked areas each contain a defect, which are shown in the inset figures (not all defects are highlighted). They reveal a number of different vacancy-type defects ranging from single vacancies (Figure 2c.5, consisting of a pentagon and a nine-atom ring with a dangling bond atom) and double vacancies of different types (Figure 2c.6–7)^17^ to more complicated structures. In addition to vacancy-type defects, also some impurity atoms are found incorporated into the material. Based on their contrast, most of these atoms are silicon, which is a typical impurity in graphene^18,19^ and has been previously introduced into vacancies from contamination.^16^

## Automatic Image Acquisition and Analysis

**Figure 3:**
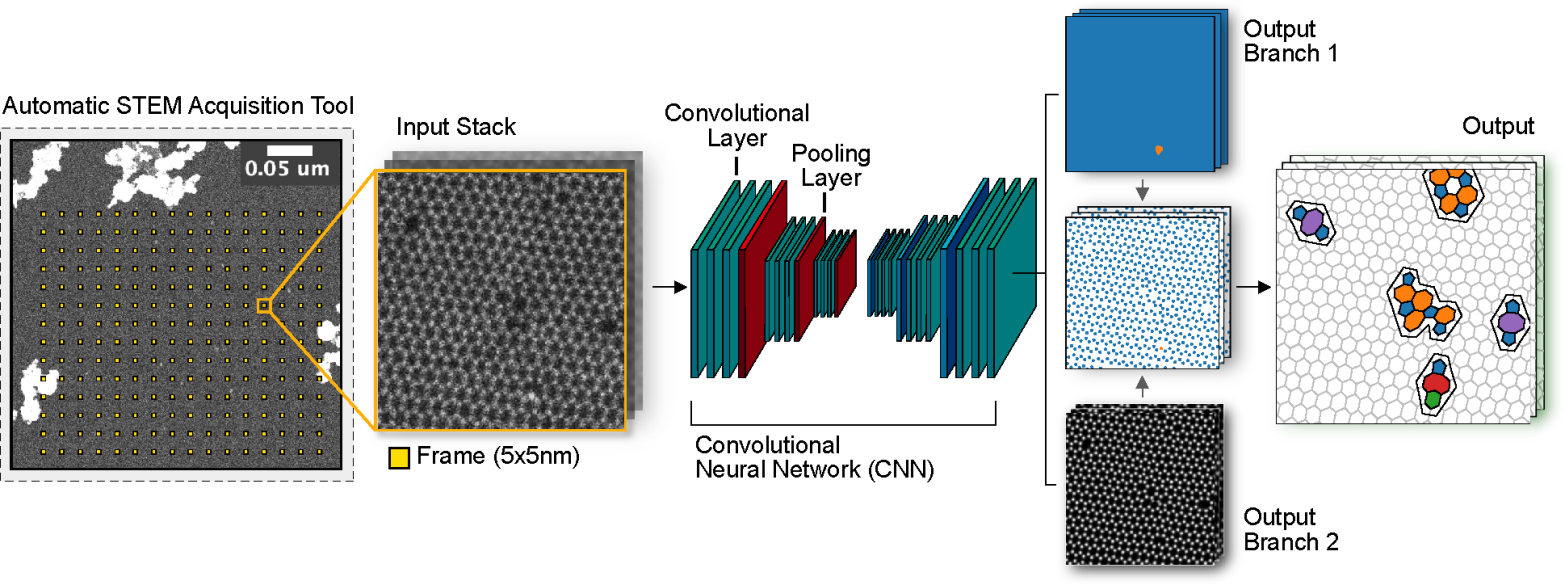
Microscopy data acquisition and analysis. First, four locations are selected to define the area to be imaged. The yellow squares on the STEM-ADF image mark the locations where the imaging algorithm recorded images (nominal field of view 5 × 5 nm^2^ with a distance of 20 nm between the images). The recorded images form a stack, which is passed onto the convolutional neural network for analysis. The CNN extracts information separately in two output branches. In the first branch, the sample composition is determined based on the image contrast, which allows distinguishing carbon atoms (blue) from other elements (orange). In the second branch, the atomic positions are detected. Information from the two branches is then combined and used as the basis for a topological analysis, which provides the final output separately for each frame.

After creating the disordered graphene structure that remains mostly free of contamination, a sufficient number of images needs to be obtained to be able to describe the sample at the atomic scale. The general working principle of our automatic image acquisition and analysis is shown in Figure 3. A database of images from a selected region of the sample is created by recording high-magnification images (a few nm^2^ field-of-view) at different locations by systematically moving the microscope stage in a serpentine path (as an example, locations of images from one such scan are shown via yellow squares in the left-most image of Figure 3). The sample height at each location is roughly estimated by a spline interpolation based on values previously recorded at the four corners of the scan map by the microscope operator. When necessary, adjustment of the electron energy (compensating for height variations) and astigmatism correction were carried out manually. Next, the database of recorded images is passed to the convolutional neural network (CNN). The CNN recognizes the atomic structure of the lattice and other features (possible remaining contaminants, pores, or unsharp image areas) separately in two output branches. In the end, these are combined to produce a topological map of the atomic structure in each image, which allows the defects to be recognized based on the arrangement of carbon rings with different numbers of atoms, producing a catalog of defects in the sample. Theneural network has a UNET-type architecture,^20^ and it was trained exclusively from simulated data; this approach has been shown to be effective in earlier work.^21^

**Figure 4:**
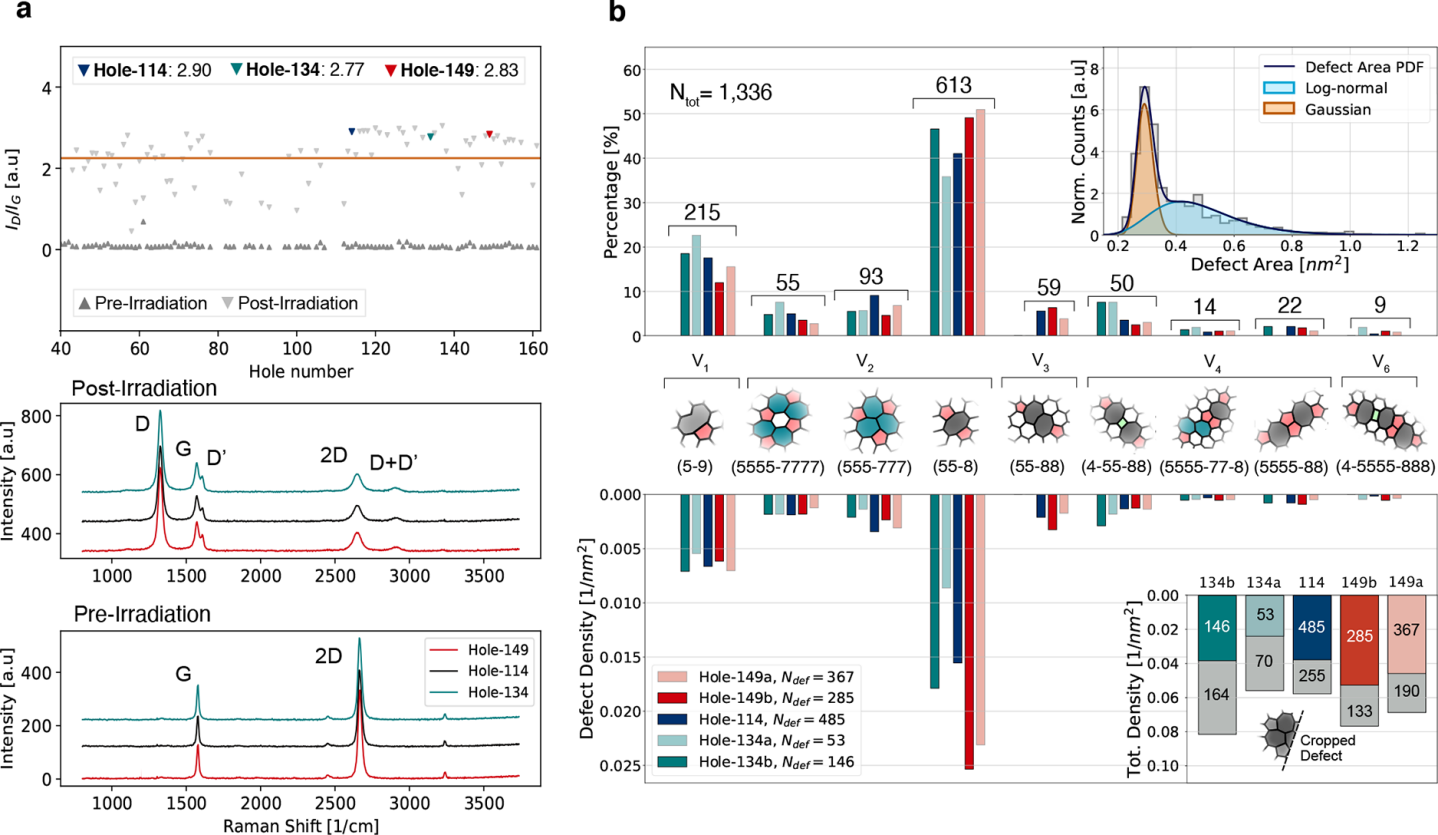
Defect density and topology. (a) *I*_D_/*I*_G_ ratios (top) for each characterized suspended graphene area before and after Ar^+^ irradiation. The results for those three holes selected for complete analysis are marked with the colored triangles, and their complete recorded spectra are shown (bottom). (b) Relative occurrence of the most often found defects, in terms of percentage and areal density, separately for each analyzed sample area. The insets show the distribution of the defect areas (top) and the total defect densities for each sample area divided into (colored bar) recognized and unrecognized (gray bar) defects. The latter category consists mostly of “cropped defects” that occurred at the edges of the image.

Specifically, we applied the method here to analyze four different sample areas. Each area contains 40 holes in the substrate, and they are located in different regions of the sample (see Supporting Information). Graphene suspended over each of the 120 holes was precharacterized via Raman spectroscopy to estimate the sample quality before plasma irradiation. The ratio of the intensities of the D and the G peaks (*I*_D_/*I*_G_) for each hole is shown in Figure 4a both before and after the irradiation (postirradiation spectroscopy measurements were carried out after the selected sample areas had been imaged to avoid removing the sample from the vacuum system before imaging). For the three holes that were selected for atomic-resolution analysis (114, 134, 149), also the complete spectra are shown. The whole sample was uniformly irradiated with Ar plasma with a current of 0.9 nA for 30 s, which results in a dose of 3.2 × 10^13^ ions cm^–2^. Before moving to the discussion of the results of the topological analysis of the sample established from the recorded images, we point out that the results of the irradiation are clearly apparent based on the postirradiation Raman spectroscopic analysis. Although some sample areas display relatively large variations in *I*_D_/*I*_G_ after irradiation (particularly holes 40–115), more uniform results were found in that sample area where holes 120–160 are located. However, the ratio increased throughout the sample compared to the preirradiation values. For the holes selected for full characterization, the values are 2.90 (hole 114), 2.83 (hole 149), and 2.77 (hole 134).

After irradiation but before the postirradiation spectroscopy analysis, microscopy images were acquired in different areas of each of the selected holes. In particular, the data collection in holes 149 and 134 consisted of two separate maps defined by an offset of 20 nm between the recorded images. Differently, for hole 114, the individual frames were separated by a distance of 5 nm. All images were recorded with 2048 × 2048 pixels. Although the 60 kV acceleration voltage used here is below the knock-on threshold in graphene,^22^ these conditions are known to be able to change the atomic configuration of defects.^23^ To avoid this, the electron dose per frame was kept relatively low (6.5 ± 1.3 × 10^6^ electrons Å^–2^ corresponding to an exposure time of 2.5 μs/pixel). Results of the topological analysis of the recorded images are summarized in Figure 4b. In total, the area characterized via microscopy was nearly 10 μm^2^, of which 0.3 μm^2^ was imaged at atomic resolution. Within this area, 1336 defects were detected by the CNN and classified according to their configuration and number of missing atoms. The defect distribution is shown in Figure 4b for each data set in terms of both percentage and areal density. At almost 50%, divacancy *V*_2_(55–8) represents the most frequent defect, followed by the single vacancy *V*_1_(5–9). The defect naming scheme used here denotes the order of the vacancy in the subscript, and the numbers in parentheses list all nonhexagonal carbon rings in order from the smallest to the largest ones.

Comparing the data sets recorded in different areas shows that the defect populations are similar throughout the characterized area. Overall, vacancies with an even number of missing atoms are clearly more prevalent than odd-numbered vacancies. Specifically, quadruple vacancies are ca. 45% more likely to occur than triple vacancies (ca. 6.4% vs 4.4% of all defects), and there are ca. 3.5 times more double vacancies than single vacancies. We point out that *V*_2_(55–8) occurs in ca. 81% of all divacancy configurations, in contrast to ca. 50% in ref (7), where transformation of the defect was followed under the electron beam, showing that the electron dose used here indeed hindered structural transformation compared to what happens under regular imaging conditions. Defect density (σ) was calculated independently for each data set based on the number of detected defects and the actual imaged sample area. For the first data set of hole 134 (134-a), the results differ slightly from the other data sets, which is caused by the somewhat smaller field of view of the images (nominally 4 vs 5 nm^2^). This leads to a larger fraction of the defects to appear at image edges being partially outside, which resulted in cropped images (contributing to a larger relative number of unrecognized defects).

The results allow us to calculate the average defect density, average distance between the defects, and their size distribution (inset in Figure 4b). Because of the minimal deviation in the *I*_D_/*I*_G_ ratio, the results of all fully characterized areas can be summarized to compare to the model proposed by Lucchese et al.^12^ The defect density is approximately 0.054 nm^–2^ (in contrast to the estimated irradiation dose of 0.32 ions nm^–2^) with on average 4.36 nm distance between the defects. The area enclosed within agglomerates of non-six-membered carbon rings provides a measure of their size (for example, the area of a *V*_2_(55–8) divacancy is equal to that of eight hexagonal carbon rings). The probability density function (Figure 4b, top inset) representing the defect area is a convolution of two modes: a (roughly) normal distributed population of small and partially mobile *V*_1_(5–9) and *V*_2_(55–8) defects with an average area of 0.31 nm^2^ and a log-normal tail that consists of both large and relatively immobile *V*_2_-type defects (such as for instance *V*_2_(55–77) and *V*_2_(555–777)) and the agglomerates of the mobile defects (monovacancies have been estimated to have a migration barrier of ca. 1.3 eV^24^) with a geometric mean of 0.42 nm^2^. We believe that the agglomerates of the mobile defects have resulted from a Brownian motion-like random walk,^25^ thermally excited by the laser irradiation. Since the increase in size reduces the mobility of the defects and slows down their further expansion, the log-normal size distribution of agglomerates emerges naturally without additional assumptions. Hence, the ion irradiation has mostly created single and double vacancies, as is expected for low-energy Ar^+^ ions.^15^

It is worth pointing out that the measured defect density is significantly lower than what was expected based on the ion current and the irradiation time. This is due to both agglomeration of the defects, as discussed above, and presumably petering ion current during the irradiation itself, pointing to the importance of measuring the defect density directly. The averaged *I*_D_/*I*_G_ value of 2.83 ± 0.06 at the measured defect density (σ = 1/*L*_D_^2^ ≈ 0.054 nm^–2^, where *L*_D_ ≈ 4.36 nm is the average distance between defects) agrees well with the value (4.53 nm) based on the model in ref (12), although the here measured defect area is much smaller than what was assumed in their phenomenological model (a defect radius of 1 nm).

## Outlook

We demonstrated structural engineering and atomic-scale analysis of graphene up to a level that has not been hitherto possible. This was made possible through a combination of three separate but equally important advances, namely: (1) an integrated (nearly) ultrahigh vacuum system that allows atomic-scale scanning transmission electron microscopy, sample cleaning, and structural manipulation without exposing the sample in between to ambient, (2) automated image acquisition that makes it possible to record a large number of images of the sample covering much larger areas than what is typically imaged, and (3) convolutional neural network trained to reliably recognize atomic structure from microscopy images to deduce the topology of defected areas. Graphene was chosen as the example material due to the general interest in tailoring its properties through structural engineering and because defected graphene has a very prominent Raman spectroscopy fingerprint that allows a direct comparison of the fully characterized structurally engineered structure to earlier research relying on this fingerprint. However, none of the three methods demonstrated here is limited to graphene and could equally well be applied to any sample suitable for transmission electron microscopy characterization.

## Supporting Information Available

Sample fabrication, Raman spectroscopy, sample cleaning, plasma irradiation, scanning transmission electron microscopy, convolutional neural network analysis, Table S1 and Figures S1–S9. Ref (15, 20, and 26−35) are cited in the Supporting Information. Original data used in the manuscript is available through the University of Vienna repository Phaidra^36^ (PDF)

## References

[ref1] Krivanek O. L.; Corbin G. J.; Dellby N.; Elston B. F.; Keyse R. J.; Murfitt M. F.; Own C. S.; Szilagyi Z. S.; Woodruff J. W. An electron microscope for the aberration-corrected era. Ultramicroscopy 2008, 108, 179–195. 10.1016/j.ultramic.2007.07.010. 18054168

[ref2] Huang P. Y.; Kurasch S.; Alden J. S.; Shekhawat A.; Alemi A. A.; McEuen P. L.; Sethna J. P.; Kaiser U.; Muller D. A. Imaging Atomic Rearrangements in Two-Dimensional Silica Glass: Watching Silica’s Dance. Science 2013, 342, 224–227. 10.1126/science.1242248. 24115436

[ref3] Toh C.-T.; Zhang H.; Lin J.; Mayorov A. S.; Wang Y.-P.; Orofeo C. M.; Ferry D. B.; Andersen H.; Kakenov N.; Guo Z.; et al. Synthesis and properties of free-standing monolayer amorphous carbon. Nature 2020, 577, 199–203. 10.1038/s41586-019-1871-2. 31915396

[ref4] Novoselov K. S. Electric Field Effect in Atomically Thin Carbon Films. Science 2004, 306, 666–669. 10.1126/science.1102896. 15499015

[ref5] Åhlgren E. H.; Kotakoski J.; Lehtinen O.; Krasheninnikov A. V. Ion irradiation tolerance of graphene as studied by atomistic simulations. Appl. Phys. Lett. 2012, 100, 233108 10.1063/1.4726053.

[ref6] Kotakoski J.; Krasheninnikov A. V.; Kaiser U.; Meyer J. C. From Point Defects in Graphene to Two-Dimensional Amorphous Carbon. Phys. Rev. Lett. 2011, 10.1103/PhysRevLett.106.105505. 21469806

[ref7] Kotakoski J.; Brand C.; Lilach Y.; Cheshnovsky O.; Mangler C.; Arndt M.; Meyer J. C. Toward two-dimensional all-carbon heterostructures via ion beam patterning of single-layer graphene. Nano Lett. 2015, 15, 5944–5949. 10.1021/acs.nanolett.5b02063. 26161575 PMC4566131

[ref8] Bangert U.; Pierce W.; Kepaptsoglou D. M. D.; Ramasse Q. M.; Zan R.; Gass M.; Van Den Berg J. A.; Boothroyd C.; Amani J.; Hofsaess H. C. Ion implantation of graphene - towards IC compatible technologies. Nano Lett. 2013, 13, 4902–4907. 10.1021/nl402812y. 24059439

[ref9] Susi T.; Hardcastle T. P; Hofsass H.; Mittelberger A.; Pennycook T. J; Mangler C.; Drummond-Brydson R.; Scott A. J; Meyer J. C; Kotakoski J. Single-atom spectroscopy of phosphorus dopants implanted into graphene. 2D Mater. 2017, 4, 021013 10.1088/2053-1583/aa5e78.

[ref10] Tapasztó L.; Dobrik G.; Nemes-Incze P.; Vertesy G.; Lambin P.; Biró L. Tuning the electronic structure of graphene by ion irradiation. Phys. Rev. B: Condens. Matter Mater. Phys. 2008, 78, 233407 10.1103/PhysRevB.78.233407.

[ref11] Ugeda M. M.; Fernández-Torre D.; Brihuega I.; Pou P.; Martínez-Galera A. J.; Pérez R.; Gómez-Rodríguez J. M. Point defects on graphene on metals. Phys. Rev. Lett. 2011, 107, 116803 10.1103/PhysRevLett.107.116803. 22026692

[ref12] Lucchese M.; Stavale F.; Ferreira E.; Vilani C.; Moutinho M.; Capaz R. B.; Achete C.; Jorio A. Quantifying ion-induced defects and Raman relaxation length in graphene. Carbon 2010, 48, 1592–1597. 10.1016/j.carbon.2009.12.057.

[ref13] Hong J.; Park M. K.; Lee E. J.; Lee D.; Hwang D. S.; Ryu S. Origin of New Broad Raman D and G Peaks in Annealed Graphene. Sci. Rep. 2013, 3, 3 10.1038/srep02700. PMC3776959 24048447

[ref14] Tripathi M.; Mittelberger A.; Mustonen K.; Mangler C.; Kotakoski J.; Meyer J. C.; Susi T. Cleaning graphene: Comparing heat treatments in air and in vacuum. Phys. Status Solidi RRL 2017, 11, 1700124 10.1002/pssr.201700124.

[ref15] Lehtinen O.; Kotakoski J.; Krasheninnikov A.; Tolvanen A.; Nordlund K.; Keinonen J. Effects of ion bombardment on a two-dimensional target: Atomistic simulations of graphene irradiation. Phys. Rev. B: Condens. Matter Mater. Phys. 2010, 81, 153401 10.1103/PhysRevB.81.153401.

[ref16] Inani H.; Mustonen K.; Markevich A.; Ding E.-X.; Tripathi M.; Hussain A.; Mangler C.; Kauppinen E. I.; Susi T.; Kotakoski J. Silicon Substitution in Nanotubes and Graphene via Intermittent Vacancies. J. Phys. Chem. C 2019, 123, 13136 10.1021/acs.jpcc.9b01894. PMC6539548 31156738

[ref17] Kotakoski J.; Meyer J.; Kurasch S.; Santos-Cottin D.; Kaiser U.; Krasheninnikov A. Stone-Wales-type transformations in carbon nanostructures driven by electron irradiation. Phys. Rev. B: Condens. Matter Mater. Phys. 2011, 83, 245420 10.1103/PhysRevB.83.245420.

[ref18] Zhou W.; Kapetanakis M.; Prange M.; Pantelides S.; Pennycook S.; Idrobo J.-C. Direct Determination of the Chemical Bonding of Individual Impurities in Graphene. Phys. Rev. Lett. 2012, 109, 206803 10.1103/PhysRevLett.109.206803. 23215517

[ref19] Ramasse Q. M.; Seabourne C. R.; Kepaptsoglou D.-M.; Zan R.; Bangert U.; Scott A. J. Probing the Bonding and Electronic Structure of Single Atom Dopants in Graphene with Electron Energy Loss Spectroscopy. Nano Lett. 2013, 13, 4989–4995. 10.1021/nl304187e. 23259533

[ref20] Ronneberger O.; Fischer P.; Brox T. U-Net: Convolutional Networks for Biomedical Image Segmentation In Lecture Notes in Computer Science (including subseries Lecture Notes in Artificial Intelligence and Lecture Notes in Bioinformatics); Springer Verlag, 2015; Vol. 9351, pp 234–241.

[ref21] Ziatdinov M.; Dyck O.; Maksov A.; Li X.; Sang X.; Xiao K.; Unocic R. R.; Vasudevan R.; Jesse S.; Kalinin S. V. Deep Learning of Atomically Resolved Scanning Transmission Electron Microscopy Images: Chemical Identification and Tracking Local Transformations. ACS Nano 2017, 11, 12742–12752. 10.1021/acsnano.7b07504. 29215876

[ref22] Susi T.; Hofer C.; Argentero G.; Leuthner G. T.; Pennycook T. J.; Mangler C.; Meyer J. C.; Kotakoski J. Isotope analysis in the transmission electron microscope. Nat. Commun. 2016, 7, 13040 10.1038/ncomms13040. 27721420 PMC5476802

[ref23] Kotakoski J.; Brand C.; Lilach Y.; Cheshnovsky O.; Mangler C.; Arndt M.; Meyer J. C. Toward Two-Dimensional All-Carbon Heterostructures via Ion Beam Patterning of Single-Layer Graphene. Nano Lett. 2015, 15, 5944–5949. 10.1021/acs.nanolett.5b02063. 26161575 PMC4566131

[ref24] Wu L.; Hou T.; Li Y.; Chan K. S.; Lee S.-T. First-Principles Study on Migration and Coalescence of Point Defects in Monolayer Graphene. J. Phys. Chem. C 2013, 117, 17066 10.1021/jp405130c.

[ref25] Kotakoski J.; Mangler C.; Meyer J. C. Imaging atomic-level random walk of a point defect in graphene. Nat. Commun. 2014, 5, 3991 10.1038/ncomms4991. 24874455 PMC4050261

[ref26] Hotz M. T.; Corbin G. J.; Dellby N.; Krivanek O. L.; Mangler C.; Meyer J. C. Ultra-High Vacuum Aberration-Corrected STEM for in-situ studies. Microsc. Microanal. 2016, 22, 34–35. 10.1017/S1431927616001021.

[ref27] Weiler M.; Cesa G. General E(2)-Equivariant Steerable CNNs. In Advances in Neural Information Processing Systems; Wallach H., Larochelle H., Beygelzimer A., Alché-Buc F. d., Fox E., Garnett R., Eds.; Curran Associates, Inc., 2019; Vol. 32.

[ref28] Paszke A.; Gross S.; Massa F.; Lerer A.; Bradbury J.; Chanan G.; Killeen T.; Lin Z.; Gimelshein N.; Antiga L. PyTorch: An Imperative Style, High-Performance Deep Learning Library. In Advances in Neural Information Processing Systems; Wallach H., Larochelle H., Beygelzimer A., Alché-Buc F. d., Fox E., Garnett R., Eds.; Curran Associates, Inc., 2019; Vol. 32, pp 8026–8037.

[ref29] Alex Kendall V. B.; Cipolla R. Bayesian SegNet: Model Uncertainty in Deep Convolutional Encoder-Decoder Architectures for Scene Understanding. In Proceedings of the British Machine Vision Conference (BMVC); Tae-Kyun Kim G. B., Stefanos Z., Mikolajczyk K., Eds.; BMVA Press, 2017; pp 57.1–57.12.

[ref30] Stuart S. J.; Tutein A. B.; Harrison J. A. A reactive potential for hydrocarbons with intermolecular interactions. J. Chem. Phys. 2000, 112, 6472–6486. 10.1063/1.481208.

[ref31] Plimpton S. Fast parallel algorithms for short-range molecular dynamics. J. Comput. Phys. 1995, 117, 1–19. 10.1006/jcph.1995.1039.

[ref32] Hjorth Larsen A.; Jorgen Mortensen J.; Blomqvist J.; Castelli I. E.; Christensen R.; Dulak M.; Friis J.; Groves M. N.; Hammer B.; Hargus C.; et al. The atomic simulation environment - A Python library for working with atoms. J. Phys.: Condens. Matter 2017, 29, 273002 10.1088/1361-648X/aa680e. 28323250

[ref33] Kirkland E. J. Advanced computing in electron microscopy, 2nd ed.; Springer US: Boston (MA), 2010.

[ref34] Madsen J.; Susi T. abTEM: ab Initio Transmission Electron Microscopy Image Simulation. Microsc. Microanal. 2020, 26, 448–450. 10.1017/S1431927620014701.

[ref35] Agarwal P. K.; Gao J.; Guibas L. J.; Kaplan H.; Rubin N.; Sharir M. Stable Delaunay Graphs. Discrete and Computational Geometry 2015, 54, 905–929. 10.1007/s00454-015-9730-x.

[ref36] Original data is available on the University of Vienna repository Phaidra. https://phaidra.univie.ac.at/o:1185095.

