# Supplementary material for: Atomic-Level Structural Engineering of Graphene on a Mesoscopic Scale
Source: Nano Lett. 2021 Jun 9;21(12):5179–85. doi: 10.1021/acs.nanolett.1c01214 (PMC8227467; doi:10.1021/acs.nanolett.1c01214)
Supplement: Supplementary file 1 — nl1c01214_si_001.pdf [file nl1c01214_si_001.pdf]

Supporting Information for

# Atomic-level structural engineering of graphene on a mesoscopic scale

Alberto Trentino,[1*] Jacob Madsen,[1] Andreas Mittelberger[2],
Clemens Mangler[1], Toma Susi[1], Kimmo Mustonen[1], Jani Kotakoski[1†]

[1]University of Vienna, Faculty of Physics, Boltzmanngasse 5, 1090 Vienna, Austria
[2]Nion Company, 11511 NE 118th Street, Kirkland, Washington 98034, USA

[*]E-mail: alberto.trentino@univie.ac.at [†]E-mail: jani.kotakoski@univie.ac.at

May 11, 2021

## Experimental Procedure

**Sample Fabrication** Commercial monolayer graphene ("Easy Transfer Graphene", Graphenea Inc., grown via chemical vapor deposition on a Cu substrate) was transferred onto a holey silicon nitride support film (PELCO, hole diameter of $2.5$ $\mu$m). Before graphene transfer, an amorphous Quantifoil carbon film was deposited on top of the SiN film acting as a marker grid to allow identifying the same sample areas in different instruments. During the transferring procedure, the material is first detached from a polymer supporting film through its immersion in deionized water. The floating material covered by a protecting sacrificial layer is fished up with the silicon nitride grid. The adhesion of the transferred material is favored by a subsequent annealing for 1 h at $150°$C followed by storage for 24 h in vacuum. The protective sacrificial-layer was removed from graphene using a hot acetone bath ($1.5$ h at $50°$C), followed by a wash in isopropyl alcohol ($1.5$ h, room temperature). Optical microscopy images of the sample are shown in Fig. S1.

**Raman Spectroscopy** Estimations of the sample quality and pre- and post-irradiation defect density were carried out through Raman spectroscopy using a combined Raman-Atomic Force Microscopy instrument WiTEC alpha 300. The individual spectra were acquired by focusing a $5$ mW laser with a wavelength of $532$ nm through a $100\times$ objective. The raw data were collected as accumulations of $60 \times 0.5$ s spectra. The analysis of the spectra was carried out through a Python routine including background subtraction and baseline correction through asymmetric least squares smoothing ($\lambda = 10$, asymmetry parameter $p = 10^{-8}$), which led to a good signal-to-noise ratio without introducing artefacts. The Whitaker-Hayes algorithm was further implemented for fixing spectra affected by cosmic-ray spikes. All peaks were afterwards fitted with single Lorentzian functions to estimate their intensities for calculating the $I_D/I_G$ ratios.

**Sample Cleaning** Upon introduction into the vacuum system, the sample is annealed at $180°$C overnight, which removes water and any weakly bound contamination. After this, the remaining chemical residues of the transfer process, as well as hydrocarbon molecules deposited onto the sample after preparation, are removed using laser irradiation in the vacuum system. One laser ($445$ nm with a tunable power up to $6$ W) is installed next to the Nion UltraSTEM 100 microscope column with optical access to the sample in the objective area through a viewport (with a rectangular spot size of ca. $0.3$ mm $\times$ $1.5$ mm), and is used for cleaning the samples prior to irradiation. Another laser of the same type is installed at the chamber containing the microwave plasma generator used for ion irradiation, and has a similar optical access to the sample through a viewport. This laser is operated continuously during the irradiation process which prevents contamination buildup on the sample. $100$ ms laser pulses were used typically at a $600$ mW power to pre-clean the samples in the microscope column, whereas the plasma chamber laser was operated at a $60$ mW root-mean-square power in continuous mode. Example images of the pre-irradiation cleaning results are shown in Fig. S2.

**Plasma irradiation** Defects were introduced into the graphene lattice in a separate vacuum chamber connected to the microscope via a direct vacuum transfer line with a pressure of ca. $10^{-8}$ mbar. The chamber itself has a typical base pressure of $5 \times 10^{-10}$ mbar. $Ar^+$ ions are created using a magnetron plasma generator with an opening towards the sample. Prior to inserting the sample, the ion current is measured with a Faraday cup. For these experiments, an irradiation time of $180$ s was used at an estimated initial beam current of $9.05$ nA, resulting in an estimated ion dose of ca. $3.2 \times 10^{13}$ ions cm$^{-2}$. No additional acceleration was applied to the ions, which are estimated by the manufacturer to have an intrinsic kinetic energy of ca. $25$ eV due to the plasma sheath potential. Such irradiation is expected to produce mainly single and double vacancies [1], as also observed here.

**Scanning transmission electron microscopy** Microscopy analysis was carried out using the dedicated STEM instrument Nion UltraSTEM 100. The instrument in Vienna has been modified from the standard setup by extending the objective area by a factor of two, allowing the viewport for the laser, and a stage that can accept sample carriers from the attached vacuum setup [2]. Typical pressure in the sample area of the instrument is ca. $2 \times 10^{-10}$ mbar. All images were recorded using a primary beam energy of $60$ keV with the medium angle annular dark field detector with collection angles of $60 - 200$ mrad. The convergence semi-angle of the electron probe was $30$ mrad. The typical beam current of the instrument is ca. $25$ pA. Sets of images were collected automatically using a self-guiding algorithm able to autonomously move the microscope stage, capture images and correct for small changes in sample height and astigmatism. To minimize the electron dose during acquisition, here these corrections were carried out manually when necessary. An initial estimate for the sample height at each location is calculated using spline interpolation based on a number of pre-set values selected by the operator at the rim of the area of interest (between four and twelve positions can be used). The area spanned by the preselected positions with known sample height is divided into a regular grid pattern where the images are recorded with the selected field of view, number of pixels and exposure time. An image is acquired at every grid position with the electron beam blanked in-between to minimize unnecessary exposure. As a final step, an overview image of the scanned area is produced, which allows correlating the individual images with the area of interest, if necessary. An example path for recording images and interpolated height values for each positions are shown in Fig. S3.

**Convolutional neural network (CNN) analysis** The convolutional neural network used to analyze the microscopy images has a UNET structure[3] with rotational equivariance following the work of Weiler *et al* [4]. The network is equivariant under operations in the cyclic group $C_8$, i.e. under rotations by $\pi/4$. The encoder part of the network consists of repeated application of two $3 \times 3$ convolutions, each followed by a rectified linear unit (ReLU) and a $2 \times 2$ max-pooling operation based on the field norm with stride 2 for downsampling. The input layer takes a greyscale image and outputs eight regular feature fields, and the number of feature fields is doubled

at every downsampling step for four steps. Note that the eight feature fields correspond to 64 feature maps in a standard CNN without $C_8$-equivariance.

The decoder part of the network is symmetric to the encoder, with the upsampling performed using bilinear interpolation, followed by a $3 \times 3$ convolution. The network has two output branches from the final feature fields: A branch for predicting a single-channel atomic density map represented by Gaussians centered at each atom and a branch for pixel-wise semantic segmentation with a channel for each of the three classes "graphene," "contamination," and "dopant," see Fig. S4. Each output branch has two convolutional layers with a kernel size of $3 \times 3$ and ReLU activation followed by a group pooling and a $1 \times 1$ convolution; the final activation is a sigmoid function and a softmax function for the density and segmentation map, respectively.

We trained the model parameters using the ADAM optimizer. The loss function is the sum of the weighted binary cross-entropy of the density map and the categorical cross-entropy of the segmentation map. The weights of the binary cross-entropy are set to zero in regions of the training images with contamination. The training set consisted of $9,000$ synthetic images of size $512 \times 512$ randomly cropped to $256 \times 256$ pixels (or $25.6 \times 25.6$ Ångström) with a batch size of $8$. The model was implemented using the Pytorch library[5], and the e2cnn library was used for rotational equivariance[4]. The learning curve is shown in Fig. S5. We see that most of the learning happens during the first epoch, and it is already stabilized within the first 8 epochs. Fig. S5 shows the precision and recall for detection of atomic positions within a threshold distance of $0.2$ Å, we see that the method reaches better than 99.9 % precision and 99.8 % recall within the first few epochs. The remaining errors after 8 epochs are generally found in regions that are highly degenerated by noise or close to the boundary of a contaminated area, defects that would not have been identified by a human expert. Some example predictions from experimental data is shown in Fig. S6.

*Pre- and post-processing*—The neural network is strictly trained for images with a pixel size of $0.1$ Å; hence, images with different pixel sizes are resized. The scaling factor is determined from the graphene lattice using a robust automated Fourier space algorithm. The image mean and standard deviation are normalised. However, in images with significant contamination, the statistics may be severely skewed due to the contrast difference between lattice and contamination. To mitigate this, we use the neural network twice; in the first pass, the segmentation map is used to create a mask for removing the contamination from the statistics. The second pass uses the image with improved normalisation for the final prediction. This could be continued recurrently until convergence; however, we find that two passes are generally enough.

A set of discrete points is derived from the density map using an algorithm similar to non-maximum-suppression for bounding boxes, adjusted to work for points. The position of the maximum pixel value of the density map is marked as a point, and the pixels within $\sigma$ from this pixel are set to zero; this is repeated in a loop until all pixel values are less than a threshold $t$. We use a threshold $t = 0.5$ and a distance between detection $\sigma = 0.4$ Å.

To assign a discrete class to each point, the segmentation map within a distance $\sigma$ of that point is summed, and the class corresponding to the channel with the largest sum is assigned.

The sum is weighted by the density map.

*Uncertainty estimation and data rejection*—The automated analysis outputs a result even when the data is degraded beyond interpretability, hence it is important to detect and remove such examples from the dataset. We use a semi-automatic approach for data rejection, where predictions that are determined to be uncertain are flagged for manual review. Due to the difficulty of properly calibrating uncertainties, all data thrown away in this study have been subject to a manual review. Further work could enable fully automated data rejection.

We use Monte Carlo dropout for estimating prediction uncertainties. Following Kendall *et al.* [6] we use $50\%$ dropout probability after all intermediate convolutional layers. The dropout is applied to the equivariant fields and not on single channels. We aggregate results of $20$ predictions at inference time in order to generate the density and segmentation map, as well as an uncertainty map for each.

There are two main sources of increased uncertainty: intermittent imaging issues from vibration and voltage spikes or structural changes of the sample in the middle of a scan line acquisition, see Fig. S5. The uncertainty of the density is also large in contaminated regions; however, these regions are removed using the segmentation map. Images with high uncertainty due to imaging issues are rejected, whereas defects with uncertainty due to structure changes are only removed from the detailed polygon defect analysis.

*Procedural generation of synthetic training data*—We generate random, physically inspired models of graphene with defects and contamination using a multistep procedure (see Fig. S6). The procedure starts from a pristine graphene sheet; disorder is introduced by randomly rotating bonds by 90 degrees or removing an atom. The modification is accepted or rejected through the Metropolis acceptance probability

$$p = \min[1, \exp[(E_{old} - E_{new})/k_B T]],$$

where $E_{old}$ and $E_{new}$ are the energies of the system before and after the proposed modification, $k_B$ is Boltzmann constant, and $T$ is the temperature. The system is relaxed after each modification using the AIREBO potential through LAMMPS and ASE [7, 8, 9]. The contamination density is simulated as a low-frequency random noise, and out-of-plane distortions of the graphene sheet are simulated by applying in-plane strain to all the atoms with a smooth spatial distribution. Finally, between zero and one percent, randomly chosen carbon atoms are exchanged for a heavier dopant. The images are simulated quickly by convolving a randomly generated probe with a delta function placed at the atomic positions. This approximation is well-justified for annular dark field images of 2D materials[10]. The probe is simulated with the abTEM code and includes defocus, spherical aberration, and third-order astigmatism [11]. The spherical aberration, $Cs$, is picked from a uniform distribution in the interval $[0, 50]$ $\mu$m, and the defocus is picked uniformly in the interval $[0.5\sqrt{Cs\lambda}, 1.5\sqrt{Cs\lambda}]$ Å. The magnitude of the astigmatism is picked from an exponential distribution with a scale of $0.5$ nm, and the angle is picked uniformly in $[0, 2\pi]$. To simulate scan distortions, the images are warped according to a random displacement field with a high spatial frequency in the horizontal direction and a root-mean-square picked from the interval $[0, 0.2]$ Å. Finally, Poisson noise is added to the im-

ages corresponding to a dose between $10^5$ and $10^6$ electrons Å$^{-2}$, resulting in training images that were generally more noisy than the experimental data.

*Graph analysis of defects*—Given a set of points representing graphene with defects, we construct the stable Delaunay graph of the points[12]. This construction is stable with respect to both noise and deformations of the points due to scan distortions. Furthermore, the graph is planar and hence comprised of well-defined faces. We define a point defect to be any set of connected non-hexagonal faces without dopants. We find the connected faces of a defect by constructing the dual of the stable Delaunay graph and labelling connected nodes corresponding to non-hexagonal faces by graph traversal. To determine the number of missing atoms in a point defect, we determine the path surrounding the defect in the dual graph. We then traverse the same path in a pristine reference lattice. The number of missing atoms is the difference between the atoms enclosed by the data and reference path. If the reference lattice path is not closed, the defect is not a point defect but contains an uneven number of dislocation centers. Graph analysis is shown in Fig. S7.

Table S1: Summarized results for defect density and average distance between defects. The table shows the actual scanned area, excluding the detected contamination, and the number of defects for each dataset. Those defects that were detected at image borders (partially outside the image) have been included with a weight of 0.5. $L_D$ is the average distance between the defects ($L_D = 1/\sqrt{\sigma}$, where $\sigma$ is the defect density).

| | Area [nm$^2$] | Number of defects [nm] | $L_D$ [nm] |
|---|---|---|---|
| Hole-114 | 12799 | 612 | 4.57 |
| Hole-134a | 2200 | 88 | 5 |
| Hole-134b | 3802 | 228 | 4.08 |
| Hole-149a | 8096 | 462 | 4.2 |
| Hole-149b | 5524 | 351 | 3.98 |

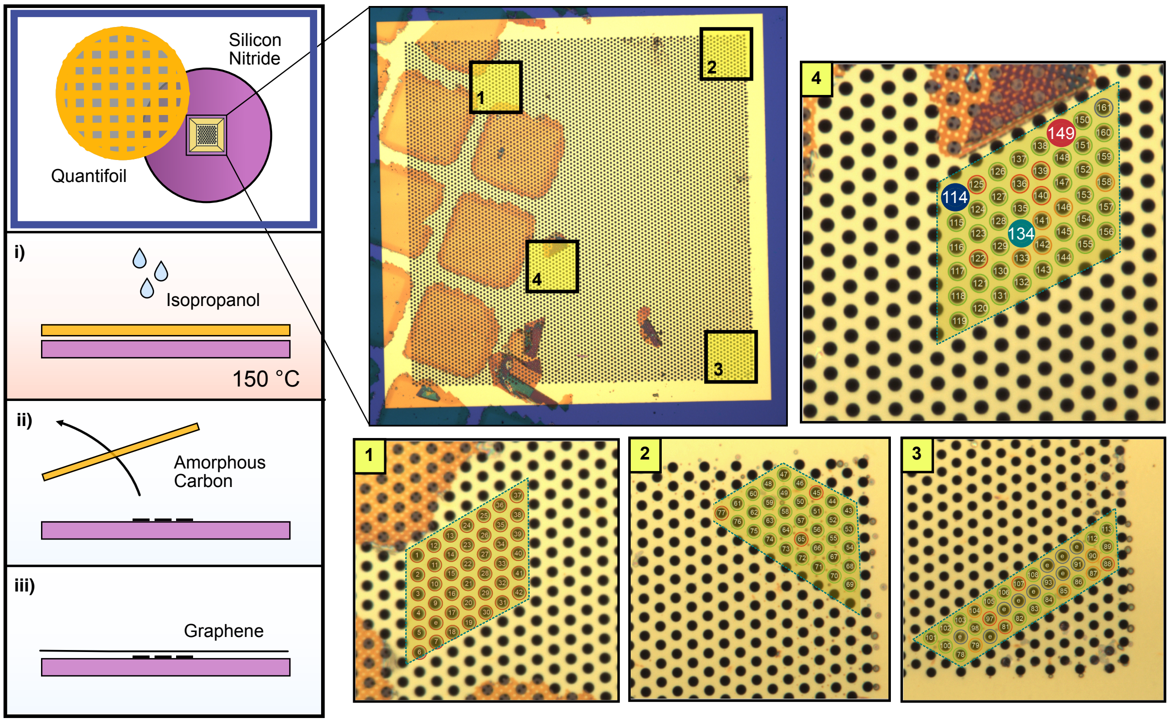


Figure S1: **Sample fabrication procedure and characterized sample areas.** (**i-iii**) A SiN grid with a holey support pattern is equipped with regions of amorphous carbon, facilitating the definition and identification of specific sample areas (1-4) throughout the experiment. These are marked in the overview optical microscopy image, and each of them is further shown at a higher magnification in the smaller labeled images. Each characterized sample area contains 40 labeled holes (empty holes are marked with ”e”).

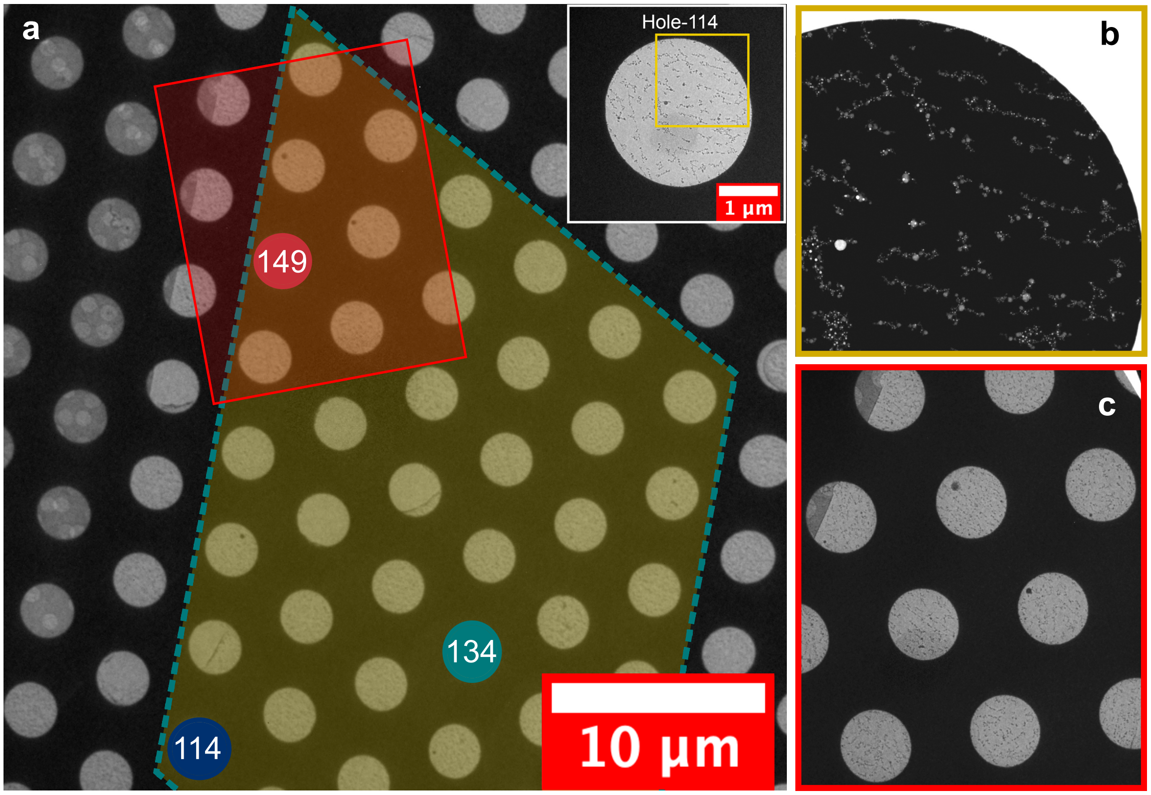


Figure S2: **Effects of laser cleaning on the micrometer scale.** (**a**) Low-magnification STEM image (recorded with the Ronchigram camera) showing results of laser cleaning at the sample area 4 (marked with the yellow overlay). The higher magnification image of hole 114 (inset) shows that only string-like features are left on suspended graphene after cleaning. (**b**) STEM-ADF image of the same hole confirms that the sample is mostly atomically clean (dark area corresponds to graphene without contamination), and that the remaining string-like features contain typically elements heavier than carbon (as can be seen from the higher contrast). (**c**) Ronchigram image of the area marked with a red rectangle in panel (A) showing that the cleaning effect extends over at least 10 $\mu$m.

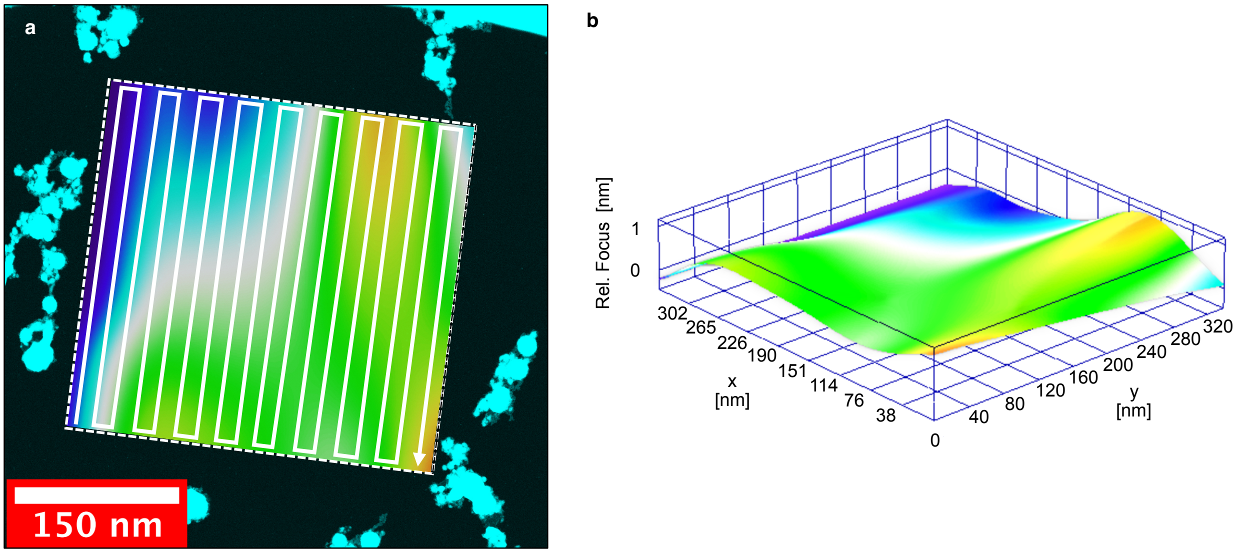


Figure S3: **Interpolation of sample height and path for automated image acquisition.** (**a**) Path taken by the algorithm while recording the first dataset (in total 305 images) for hole 149 (149-a), overlaid on the STEM-ADF overview image. The colors in this area correspond to the sample height. (**b**) Perspective plot of the sample height during the acquisition. Note that the maximum height changes within the are in the order of 1 nm.

(a) 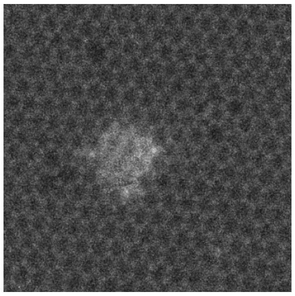 (b) 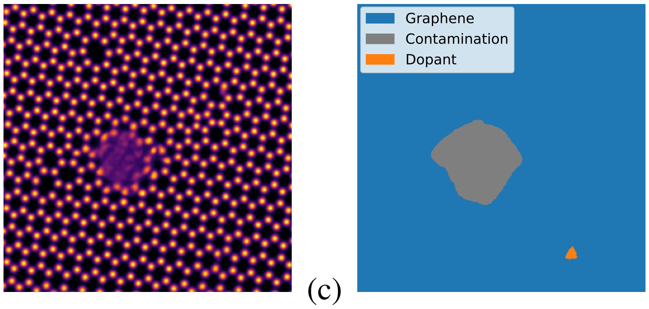 (c)


Figure S4: **Example input and output from the neural network.** (a) Input STEM image. (b) Output density of atomic positions. (c) Output pixel-wise segmentation map.

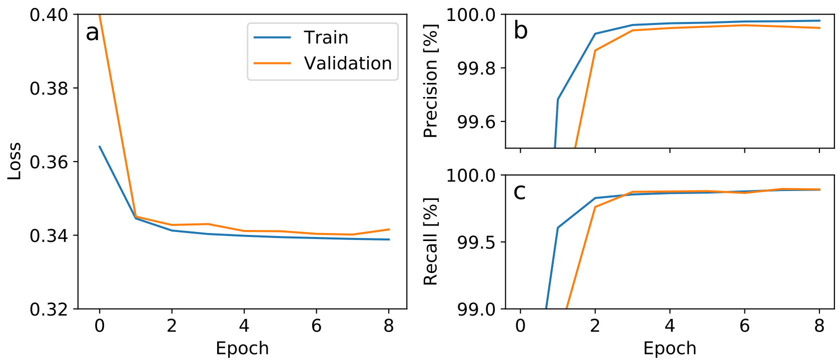


Figure S5: **Learning curves for training the neural network.** Loss (a), precision (b) and recall (c) as a function of training epoch.

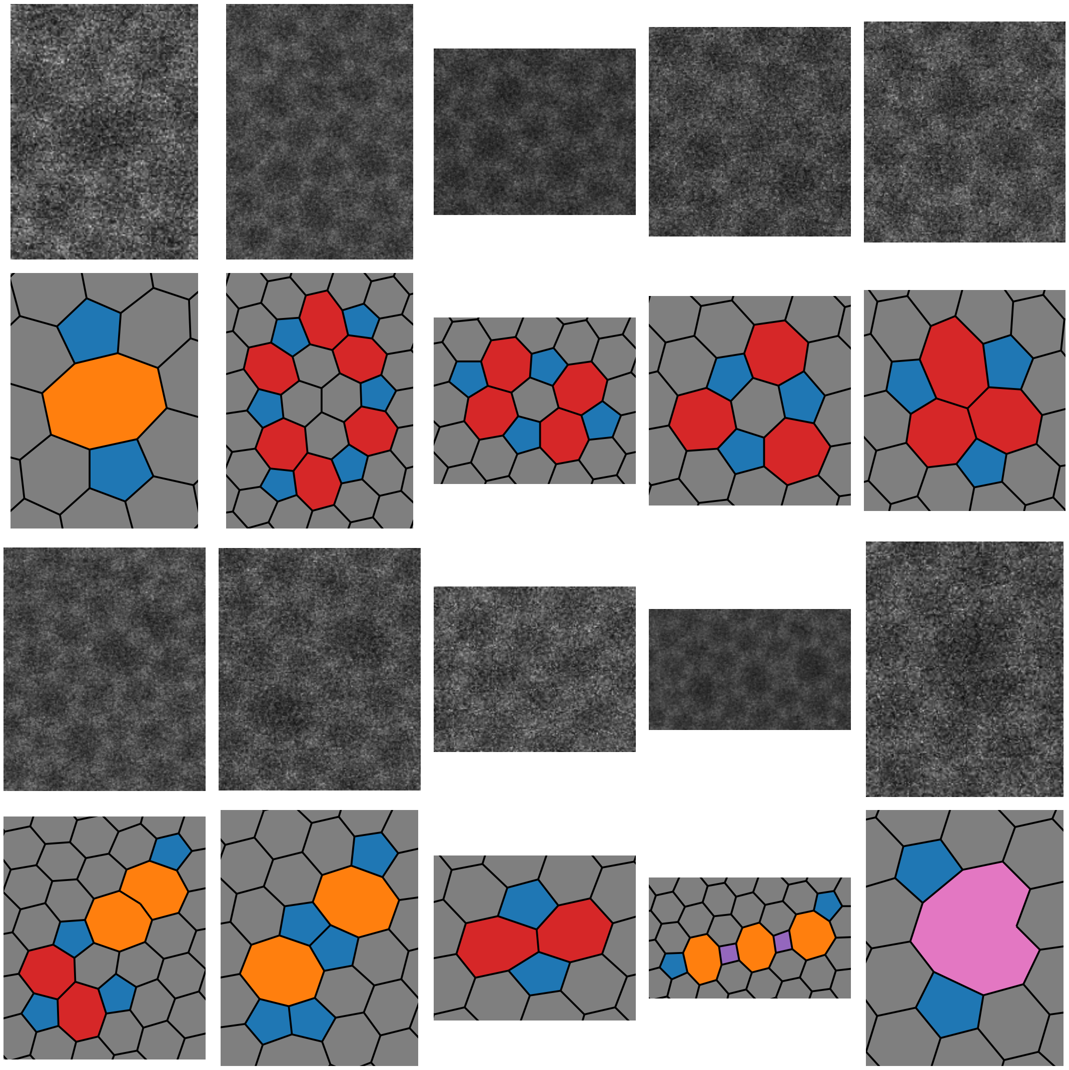

Figure S6: **Example image patches from the experimental dataset containing defects and corresponding output of the automated analysis algorithm.**

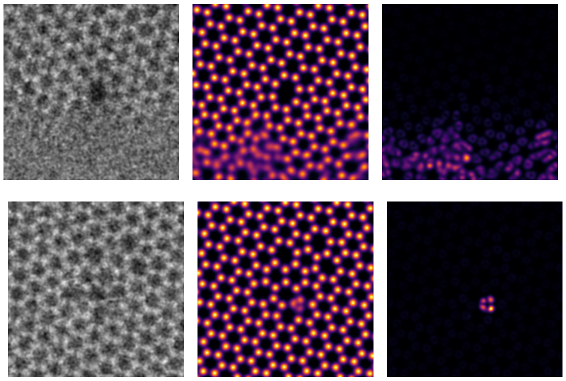

(a)

(b)

Figure S7: **Images with corresponding density maps and uncertainty estimation from Monte Carlo dropout.** (a) Uncertainty from image region that have been degraded due to a voltage spike. (b) Uncertainty from structure change in the middle of a scan line acquisition.

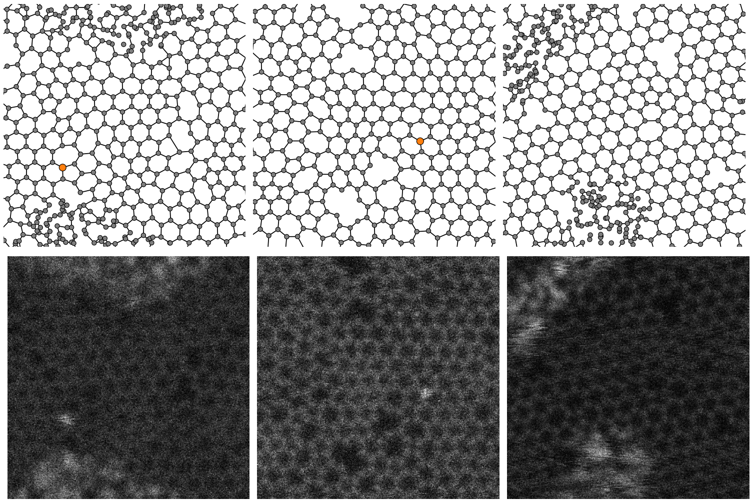

Figure S8: **Examples of randomly generated atomic structures and corresponding synthetic images used for training the neural network.**

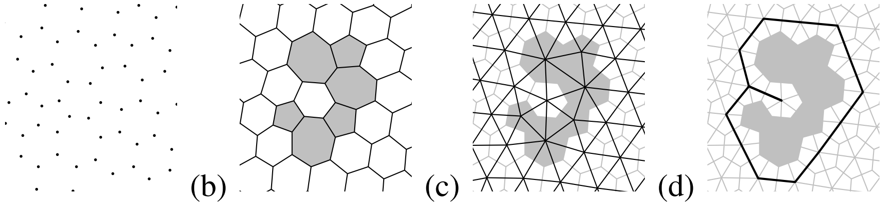

(a) (b) (c) (d)

Figure S9: **Graph analysis of a set of points to characterize defects in the lattice.** (a) A set of points describing a structure with a defect. (b) The stable Delaunay graph of the points. The non-hexagonal faces have been highlighted. (c) The dual of the stable Delaunay graph. (d) The shortest path through the hexagonal lattice surrounding a defect.

# References


[1] Lehtinen, O.; Kotakoski, J.; Krasheninnikov, A.; Tolvanen, A.; Nordlund, K.; Keinonen, J. Effects of ion bombardment on a two-dimensional target: Atomistic simulations of graphene irradiation. *Physical Review B* **2010**, 81, 153401.

[2] Hotz, M. T.; Corbin, G. J.; Dellby, N.; Krivanek, O. L.; Mangier, C.; Meyer, J. C. Ultra-High Vacuum Aberration-Corrected STEM for in-situ studies. *Microscopy and Microanalysis* **2016**, 22, 34–35.

[3] Ronneberger, O.; Fischer, P.; Brox, T. U-Net: Convolutional Networks for Biomedical Image Segmentation. In *Lecture Notes in Computer Science (including subseries Lecture Notes in Artificial Intelligence and Lecture Notes in Bioinformatics)*. Springer Verlag, volume 9351, pp. 234–241.

[4] Weiler, M.; Cesa, G. General E(2)-Equivariant Steerable CNNs. In *Advances in Neural Information Processing Systems 2019*. pp. 14334–14345.

[5] Paszke, A.; Gross, S.; Massa, F.; Lerer, A.; Bradbury, J.; Chanan, G.; Killeen, T.; Lin, Z.; Gimelshein, N.; Antiga, L.; *et al.* PyTorch: An Imperative Style, High-Performance Deep Learning Library. In Wallach, H.; Larochelle, H.; Beygelzimer, A.; Alché-Buc, F. d.; Fox, E.; Garnett, R., eds., *Advances in Neural Information Processing Systems*. Curran Associates, Inc., volume 32, pp. 8026–8037.

[6] Kendall, A.; Badrinarayanan, V.; Cipolla, R. Bayesian segnet: Model uncertainty in deep convolutional encoder-decoder architectures for scene understanding. In *British Machine Vision Conference 2017, BMVC 2017*. BMVA Press.

[7] Stuart, S. J.; Tutein, A. B.; Harrison, J. A. A reactive potential for hydrocarbons with intermolecular interactions. *Journal of Chemical Physics* **2000**, 112, 6472–6486.

[8] Plimpton, S. Fast parallel algorithms for short-range molecular dynamics. *Journal of Computational Physics* **1995**, 117, 1–19.

[9] Hjorth Larsen, A.; Jørgen Mortensen, J.; Blomqvist, J.; Castelli, I. E.; Christensen, R.; Dulak, M.; Friis, J.; Groves, M. N.; Hammer, B.; Hargus, C.; *et al.* The atomic simulation environment - A Python library for working with atoms. *Journal of Physics Condensed Matter* **2017**, 29, 273002.

[10] Kirkland, E. J. *Advanced computing in electron microscopy: Second edition*. Springer US **2010**.

[11] Madsen, J.; Susi, T. abTEM: ab Initio Transmission Electron Microscopy Image Simulation. *Microscopy and Microanalysis* **2020**, 26(S2), 448–450.

[12] Agarwal, P. K.; Gao, J.; Guibas, L. J.; Kaplan, H.; Rubin, N.; Sharir, M. Stable Delaunay Graphs. *Discrete and Computational Geometry* **2015**, 54, 905–929.
